# Delayed Skin Allergy to Glucosamine Chondroitin Supplement

**DOI:** 10.7759/cureus.36310

**Published:** 2023-03-17

**Authors:** Eric Chun-Pu Chu, Kevin Hsu Kai Huang, Gordon Cheung, Gabriel Ng, Andy Lin

**Affiliations:** 1 Chiropractic Department, New York Medical Group, EC Healthcare, Hong Kong, HKG; 2 Chiropractic Department, New York Medical Group, EC Healthcare, Yuen Long, HKG

**Keywords:** chatgpt, degenerative spine disease, chiropractic management, chiropractic therapy, glucosamine, glucosamine sulfate

## Abstract

Glucosamine chondroitin is a popular dietary supplement used for joint health and osteoarthritis pain and is one of the dietary supplements commonly recommended by chiropractors. Herein, we present the case of a 36-year-old woman who developed a skin rash with delayed onset after taking glucosamine and chondroitin pills for lumbar degenerative joint disease. Within 3 hours of taking the supplement, she developed an itchy rash on her torso and legs. Over the next few hours, the rash spread over her entire body, and facial swelling developed. Given the timing of the symptoms after the administration of glucosamine chondroitin, an allergic reaction was suspected. The supplement was withdrawn and the allergic reactions were treated with antihistamines and steroids for several days. This case report demonstrates the need to recognize delayed allergic reactions as a potential side effect of widely used supplements, such as glucosamine chondroitin, which can produce hypersensitivity reactions in sensitive individuals.

## Introduction

Non-steroidal anti-inflammatory drugs, physical therapy, massage, acupuncture, chiropractic care, and nutritional supplements such as glucosamine and chondroitin have all been employed as conservative treatments for lower back discomfort associated with degenerative joint disease [1]. Glucosamine chondroitin is a popular dietary supplement used to promote joint health and prevent osteoarthritic pain [2]. It is composed of glucosamine, an amino sugar, and chondroitin, a complex carbohydrate that is a major component of the cartilage. As a natural supplement, glucosamine chondroitin is thought to help maintain and possibly restore joint cartilage, thereby reducing pain and improving joint function [3]. Glucosamine chondroitin has been shown in the literature to delay the progression of degenerative joint diseases [4] and is one of the top-selling supplements in the U.S. for arthritis [2]. As such, reports of potential adverse reactions are observed during clinical follow-up.

While glucosamine chondroitin is generally well tolerated, adverse reactions and allergies to this supplement have previously been reported in the medical literature [5]. Epigastric discomfort (3.5%), heartburn (2.7%), diarrhea (2.5%), and nausea (1%) are the most prevalent adverse events [6]. More serious but rare adverse effects include allergic reactions which may present as itching, hives, and swelling [5]. There have also been a small number of case reports of severe allergic reactions, including anaphylaxis [5]. Further, delayed-onset allergic reactions to glucosamine chondroitin, particularly isolated skin rashes, have rarely been described. This case report details a delayed skin allergy reaction to glucosamine chondroitin in a patient who presented to a chiropractic clinic.

Chiropractors are primary care clinicians who constantly manage and diagnose arthritic pathological conditions [7]. Chiropractors undergo extensive nutritional education during chiropractic college training [8], and 72% of chiropractors recommend dietary supplements to patients with arthritic and degenerative conditions [8]. Glucosamine chondroitin is one of the most commonly recommended supplements by chiropractors [8], and this case is therefore important as it highlights the potential for an immediate and life-threatening allergic reaction to glucosamine chondroitin and its management. Although rare, patients should be informed of adverse events of glucosamine chondroitin and must be advised to seek medical help promptly if such a reaction is suspected.

## Case presentation

A 36-year-old female with no significant medical history presented to a chiropractic clinic with chronic lower back pain. The patient was diagnosed with lumbar degenerative joint disease after evaluation. As part of her treatment plan, the chiropractor recommended daily glucosamine chondroitin supplementation (1500 mg glucosamine sulfate and 1200 mg chondroitin sulfate) to help maintain joint cartilage and potentially reduce inflammation and pain. The patient has never taken glucosamine chondroitin supplements previously, and she denied any past allergic history of food or supplements.

**Figure 1:**
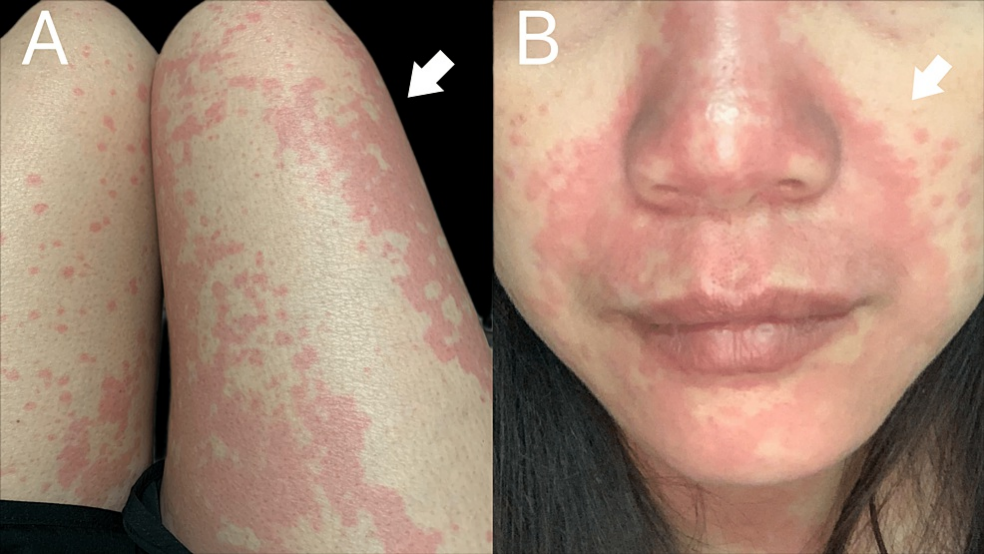
Photo of the patient after taking the supplement A) Three hours after taking the supplement, rashes appeared bilaterally on both legs, with a particularly bad rash on the right. B) Six hours after taking the supplement, rashes also developed on the nose and around the lips.

The patient took the supplement immediately after chiropractic treatment. However, within 3 hours of the first dose, she noticed the appearance of an itchy rash on her torso and legs (Figure 1A). Over the next few hours, the rash spread across her entire body, accompanied by facial swelling (Figure 1B) and stiffness in the chest. The patient also experienced nausea, dizziness, and vomiting. She called the chiropractic clinic in a state of distress and was concerned that she may be having a severe allergic reaction. The chiropractor advised her to discontinue the supplement immediately and to call emergency services or go to the nearest Emergency Department.

The patient promptly visited the Emergency Department, where she was diagnosed with anaphylaxis, likely resulting from glucosamine chondroitin supplementation. She was administered intramuscular epinephrine, antihistamines, and steroids and was admitted for monitoring. Her symptoms resolved over the next few hours, and she was discharged the following day with a prescription for an epinephrine auto-injector, and advice to avoid glucosamine chondroitin supplementation in the future. On the 14th-day follow-up phone call, she reported her symptoms recovered after 48 hours. She never experienced similar symptoms in the past two weeks.

## Discussion

The mechanism underlying delayed skin allergic reactions to glucosamine chondroitin is not yet fully understood. As a supplement derived from shellfish, shrimp, and crab exoskeletons, glucosamine chondroitin may induce an immune response and subsequent allergic reaction in individuals with a sensitivity or allergy to shellfish [5]. However, the patient in this case reported having no known shellfish allergies. Glucosamine chondroitin may also potentially interact with immune cells, such as basophils, which mediate allergic reactions, inducing the release of histamine and other inflammatory mediators that can result in a hypersensitivity rash. The components of glucosamine chondroitin supplements are complex, and the specific components responsible for adverse reactions remain unclear [5]. Glucosamine and chondroitin formulations should explicitly indicate any potential negative effects on individuals prone to shellfish allergies or atopy [5].

While glucosamine chondroitin supplements are widely used, estimates of the prevalence of allergic reactions vary. A randomized, multicenter, double-blind Brazilian study of adverse events to glucosamine and chondroitin in patients with osteoarthritis (OA) of the knee suggests that incidents are rare [9]. Another study in the United States also found similar results and reported that serious adverse events were rare over the 2-year follow-up period [10]. However, an Australian spontaneous adverse drug reaction (ADRs) study of glucosamine and chondroitin reported that 71.85% of incidents were identified as having hypersensitivity reactions, of which 16% were severe cases involving symptoms such as gait disturbance, amnesia, hypotension, and somnolence [5]. Without large-scale investigations, the prevalence of delayed skin allergy to glucosamine chondroitin will remain unknown, and it is likely that these are underestimated or misdiagnosed.

This single case report cannot reveal the true incidence or prevalence of delayed cutaneous allergic reactions to glucosamine chondroitin owing to its limitations. Additional case reports and, preferably, larger-scale investigations are needed to properly characterize this adverse reaction pattern. The mechanisms by which some individuals suffer delayed as opposed to acute hypersensitivity reactions to glucosamine chondroitin could be elucidated through additional research. Despite their rarity, the adverse effects of this widely used dietary supplement must be studied due to the ubiquity of its use. In addition, the results of such studies may have implications for enhancing product labeling, patient disclosure of risks, and professional monitoring of adverse effects when advising or prescribing glucosamine chondroitin [5].

Overall, this case reports illustrates the importance of patient education and monitoring when prescribing or recommending glucosamine chondroitin supplements. Patients should be made aware of the potential risks of adverse events such as delayed rashes and other allergic reactions. If a patient experiences suspicious symptoms that are uncommon, they should be instructed to cease using the supplement and notify their physician. Although adverse incidents in chiropractic therapy are very rare [11], chiropractors should periodically monitor patients for side effects when the supplement is first started.

**Figure 2:**
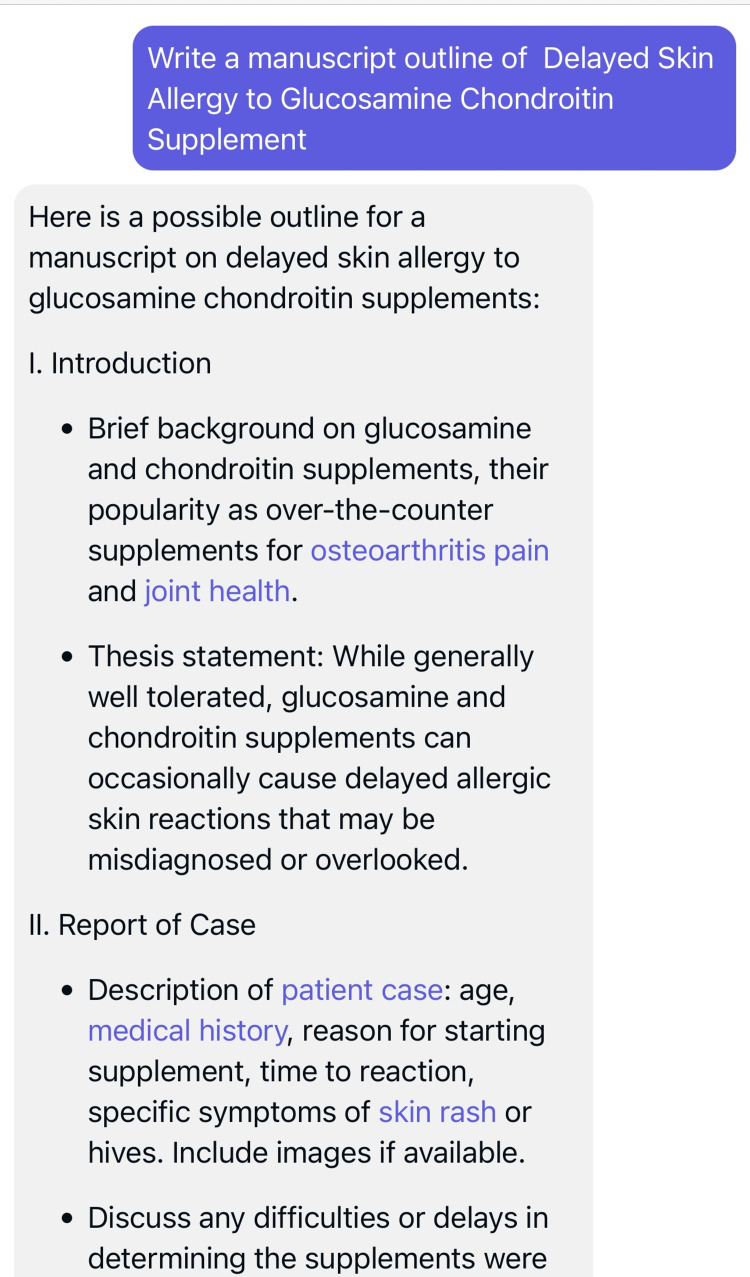
Screenshot of the utilization of ChatGPT The authors used ChatGPT in drafting the initial manuscript outline for the case report.

Our case report's outline was drafted with the assistance of ChatGPT (OpenAI Inc., San Francisco, USA) (Figure 2), and the discussion provides insights for improving the safety of this commonly used supplement.

## Conclusions

This report describes the case of a 36-year-old female who presented with a delayed-onset skin rash after treatment with glucosamine chondroitin pills. The timing and clinical manifestations of her symptoms were compatible with adverse reactions to the supplement. Several days after ceasing glucosamine chondroitin and initiating antihistamines and steroids, the rash disappeared. This example demonstrates the need to include supplement-induced delayed allergic reactions in the follow-up when patients are recommended to take supplements and experience suspicious skin rashes or other reactions. Although uncommon, delayed supplement allergies can occur and may not always be recognized. This case highlights that clinicians and patients should be aware of this potential reaction and the need to quit supplementation and seek medical assessment and treatment.
